# Health and economic effects from linking bedside and outpatient tobacco cessation services for hospitalized smokers in two large hospitals: study protocol for a randomized controlled trial

**DOI:** 10.1186/1745-6215-13-129

**Published:** 2012-08-01

**Authors:** Jeffrey L Fellows, Richard Mularski, Lisa Waiwaiole, Kim Funkhouser, Julie Mitchell, Kathleen Arnold, Sabrina Luke

**Affiliations:** 1 Kaiser Permanente Center for Health Research, 3800 N Interstate Avenue, Portland, OR 97227, USA; 2 Oregon Health & Science University, 3181 SW Sam Jackson Park Road, Portland, OR, 97239, USA

**Keywords:** Smoking cessation, Hospitalized smokers, Assisted referral to outpatient services, Cost-effectiveness analysis, Randomized clinical trial

## Abstract

**Background:**

Extended smoking cessation follow-up after hospital discharge significantly increases abstinence. Hospital smoke-free policies create a period of ‘forced abstinence’ for smokers, thus providing an opportunity to integrate tobacco dependence treatment, and to support post-discharge maintenance of hospital-acquired abstinence. This study is funded by the National Heart, Lung, and Blood Institute (1U01HL1053231).

**Methods/Design:**

The Inpatient Technology-Supported Assisted Referral study is a multi-center, randomized clinical effectiveness trial being conducted at Kaiser Permanente Northwest (KPNW) and at Oregon Health & Science University (OHSU) hospitals in Portland, Oregon. The study assesses the effectiveness and cost-effectiveness of linking a practical inpatient assisted referral to outpatient cessation services plus interactive voice recognition (AR + IVR) follow-up calls, compared to usual care inpatient counseling (UC). In November 2011, we began recruiting 900 hospital patients age ≥18 years who smoked ≥1 cigarettes in the past 30 days, willing to remain abstinent postdischarge, have a working phone, live within 50 miles of the hospital, speak English, and have no health-related barriers to participation. Each site will randomize 450 patients to AR + IVR or UC using a 2:1 assignment strategy. Participants in the AR + IVR arm will receive a brief inpatient cessation consult plus a referral to available outpatient cessation programs and medications, and four IVR follow-up calls over seven weeks postdischarge. Participants do not have to accept the referral. At KPNW, UC participants will receive brief inpatient counseling and encouragement to self-enroll in available outpatient services. The primary outcome is self-reported thirty-day smoking abstinence at six months postrandomization for AR + IVR participants compared to usual care. Additional outcomes include self-reported and biochemically confirmed seven-day abstinence at six months, self-reported seven-day, thirty-day, and continuous abstinence at twelve months, intervention dose response at six and twelve months for AR + IVR recipients, incremental cost-effectiveness of AR + IVR intervention compared to usual care at six and twelve months, and health-care utilization and expenditures at twelve months for AR + IVR recipients compared to UC.

**Discussion:**

This study will provide important evidence for the effectiveness and cost-effectiveness of linking hospital-based tobacco treatment specialists’ services with discharge follow-up care.

**Trial Registration:**

ClinicalTrials.gov: NCT01236079

## Background

How health-care facilities treat tobacco dependence in patients with cardiac and pulmonary diagnoses is a quality-of-care measure that ultimately affects whether facilities meet accreditation standards [1]. The Joint Commission recently expanded the requirement that health-care facilities also treat tobacco dependence to patients with other diagnoses, and broadened the requirements for what constitutes effective treatment [2,3]. Efforts to establish tobacco cessation as an element of treatment required to meet inpatient standards of care capitalizes on the forced abstinence that occurs with a hospital stay in a smoke-free facility. Hospital stays can also potentially increase smokers’ motivation to remain quit if their illness and hospitalization is smoking-related. Previous research has shown that initiating professional treatment during this ‘teachable moment’, and providing patients with multiple intervention contacts after discharge, leads to significant increases in quit rates [3-10]. Smokers with four or more weeks of cessation support postdischarge are more likely to be abstinent after a year compared to those without support [7].

Creating an integrated clinical pathway—from inpatient assistance to outpatient cessation services—is challenging for any health-care delivery system, even closed-model HMOs that provide inpatient services as well as outpatient clinical care and behavior-change services. An effective model includes in-hospital treatment by trained professionals whose primary responsibility is tobacco cessation [10] and a hospital-managed follow-up program for continuity of care [11]. With the growing use of electronic medical records (EMR) to support medical decisions and document care, any tobacco-cessation model also should be able to establish an integrated clinical pathway, from admission through discharge instructions and follow-up, for health providers to order and document treatment and referrals. Integrating inpatient and outpatient cessation services is much more daunting for free-standing and academic hospitals that serve patients who are potentially covered by dozens of health insurance plans. Typically, insurance plans will cover inpatient services but prior authorization is needed for outpatient coverage. Creating an effective hospital-initiated treatment program requires an innovative solution to bridge the gap from inpatient to effective outpatient care that lasts at least four weeks postdischarge.

This paper describes the protocol for the Inpatient Technology-Supported Assisted Referral (I-TSAR) study. This randomized controlled trial evaluates the effectiveness and cost-effectiveness of an approach for initiating and integrating tobacco treatment into hospital care and continuing follow-up care for patients admitted to Kaiser Permanente Northwest (KPNW) and Oregon Health & Science University (OHSU) hospitals. This intervention uses existing electronic medical records systems and tobacco treatment specialists (TTS) (nurses and/or health educators) to identify smokers and deliver effective components of hospital-initiated treatment; provide proactive assisted referrals to available outpatient counseling programs and medications; and link patients to an innovative interactive voice recognition (IVR) telephone follow-up system. IVR follow-up represents a promising method for efficient postdischarge follow-up for treatment plans initiated during hospitalization [12-14]. Combining tobacco treatment expertise with health-system technology can help address many of the existing problems with integrating in-hospital tobacco-dependence treatment and cost-effective outpatient follow-up care.

## Methods/Design

This is a multicenter, randomized usual care-controlled clinical effectiveness trial conducted with patients admitted to two large hospitals serving the Portland, Oregon and southwest Washington metropolitan area. We will randomize 900 participants to receive either an inpatient assisted referral for postdischarge tobacco-cessation services plus interactive voice recognition support (AR + IVR) or usual care (UC). This study is one of six studies funded by the National Institutes of Health (NIH) that make up the Consortium of Hospitals Advancing Research on Tobacco (CHART). The study was approved by the Institutional Review Board (IRB) of each participating institution, and all participants provide written informed consent. The CHART Data Safety Monitoring Board (DSMB) also reviewed and approved the study.

### Setting

Participants will be recruited from KPNW Sunnyside Medical Center and OHSU hospital in Portland, Oregon. KPNW is a federally qualified, not-for-profit HMO serving more than 470,000 members in northwest Oregon and southwest Washington through one hospital and 26 medical offices. It is an integrated, group-model health-delivery system that provides and coordinates the entire scope of care for its members, including access to a range of tobacco-cessation services through its Health Education Department. Existing inpatient tobacco-cessation services encourage patients with pneumonia, chronic heart failure, and ischemic heart disease to quit and enroll in cessation services upon discharge. OHSU is an academic health center serving a multistate area with specialty tertiary health-care services. OHSU has an existing inpatient tobacco-cessation consult service provided to patients referred by hospital staff: the service includes encouragement to use outpatient cessation services and a single follow-up call shortly after discharge.

KPNW and OHSU have comprehensive EMR systems and have established tobacco-free campuses. In 2010, the Oregon legislature mandated that all Oregon commercial health insurance plans provide a $500 benefit for tobacco-cessation counseling and FDA-approved medications. Oregon’s Medicaid program already provides comprehensive coverage.

### Population

The study population consists of adult patients age ≥18 years admitted to KPNW or OHSU hospitals who have smoked a cigarette (even a puff) within the past 30 days, speak English, have a working telephone, are interested in attempting to remain abstinent from smoking postdischarge, and can participate in the informed consent process. Patients must also live within 50 miles of the hospital and be willing to attend an in-person follow-up visit at six months. Patients are excluded from the study if they are admitted to a critical care, labor/delivery, or psychiatric unit, are pregnant or breastfeeding, have access restrictions (for example, MRSA), are physically too ill to participate in a research study (that is, cannot complete a six-month follow-up), or are cognitively unable to provide informed consent. Patients with a history of mental illness are included if admitted to nonpsychiatric units.

The eligibility requirements well represent the population of hospitalized current smokers and very recent quitters who meet the Joint Commission targeted patient population. The Joint Commission’s tobacco treatment standards apply to all patients regardless of diagnosis, except the cognitively impaired [2], who would likely be interested in services to help them remain abstinent after they leave the hospital, and could benefit from enrollment assistance from hospital staff and brief telephone follow-up. The exceptions to this standard include women in labor/delivery units and patients who are unlikely to attend a six-month in-person follow-up assessment visit. This and other CHART studies define smoking eligibility as having smoked at least one cigarette in the last thirty days. This commonly used definition recognizes the likelihood that many patients may report quitting smoking even though they may have stopped within the couple of weeks prior to admission. The TTS will postpone a consult visit if a patient cannot speak, is heavily medicated, in obvious pain, or has restricted access. In some instances, we expect the TTS EMR review to indicate that a patient is too physically ill to participate, for example, the patient is documented with a severely life-limiting condition or is to be discharged to hospice care.

Administrative data show that more than 5,000 current adult smokers meeting the above eligibility requirements are discharged each year from KPNW and OHSU hospitals (Table1). The demographic characteristics of discharged smokers are similar between the two sites. Ethnicity and race are routinely collected at hospital admission at both institutions, although KPNW only recently began collecting this information. In 2010, data indicate that hospitalized smokers were predominantly non-Hispanic and white. According to the 2010 Census data, about 19.5% of the Portland, Oregon metropolitan area served by Kaiser Permanente and OHSU is of either non-white or mixed race and 10.9% are of Hispanic ethnicity. Consequently, our study will over-recruit Hispanic and other minorities in order to match the demographics of the surrounding community.

**Table 1:** Characteristics of likely eligible smokers discharged from KPNW and OHSU hospitals in 2010

		**Kaiser Permanente Northwest**		**Oregon Health & Science University**	
**Patient characteristic**	**N**	**%**	**N**	**%**
**Total discharged smokers**	3401	100.0	2208	100.0
**Male**	1757	51.7	1142	51.7
**Age group**				
**18-24**	12	0.4	193	8.7
**25-34**	101	10.7	390	17.7
**35-44**	363	20.3	353	16.0
**45-54**	690	26.6	555	25.1
**55-64**	905	20.4	454	20.6
**65-74**	695	12.5	188	8.5
**75+**	211	6.2	75	3.4
**Ethnicity**				
**Hispanic/Latino**	74	2.2	77	3.5
**Non-Hispanic**	1919	56.4	2111	95.6
**Unknown**	1408	41.4	20	0.9
**Race**				
**White**	2700	79.4	1964	89.0
**Black/African American**	140	4.1	118	5.3
**Asian and Pacific Islander**	59	1.7	46	2.1
**American Indian/Alaska Native**	40	1.2	35	1.6
**Other**	32	0.9	24	1.1
**Unknown**	357	10.5	21	1.0
**Insurance plan type**				
**Commercial**	1899	55.8	578	26.2
**Medicare**	1264	37.2	554	25.1
**Medicaid/OHP**	95	2.8	645	29.2
**Nonsponsored**	101	3.0	332	15.0
**Other sponsored**	42	1.2	99	4.5

Discharge data also show that smokers were somewhat more likely to be male. Thus, our recruitment strategy incorporates a 50% female/male enrollment target.

### Recruitment

**Figure 1:**
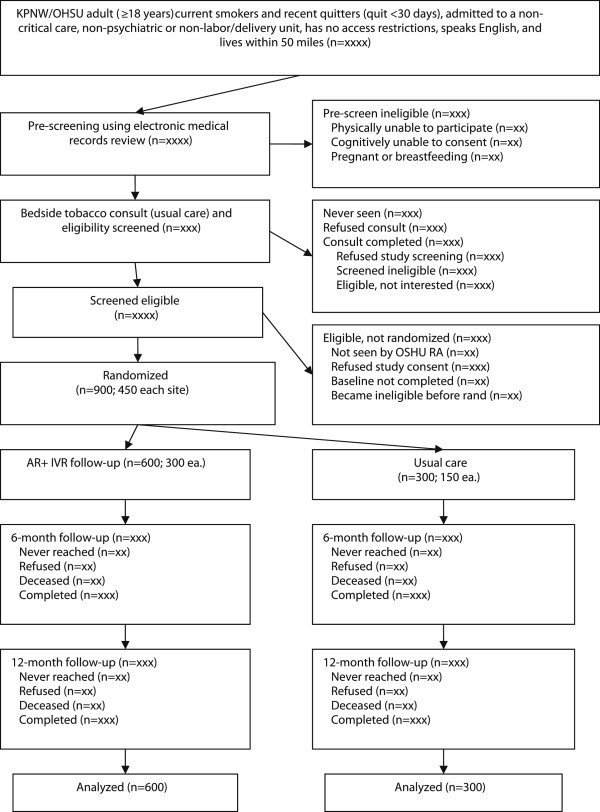
I-TSAR consort diagram.

Participant recruitment involves an initial prescreening process using electronic administrative and medical records data at each site, followed by an in-person screening by the TTS at each site for patients who appear eligible during EMR prescreening. The EMR prescreening process allows the TTS to efficiently identify likely eligible patients and review relevant medical histories before an in-person consultation. The recruitment process is reflected in the I-TSAR consort diagram (Figure1).

We will need to recruit about 12% of the estimated 6,250 adult smokers who meet our eligibility criteria in order to meet our recruitment target of 900 smokers over 15 months. Our experience with patient populations suggests that 50% or more of identified smokers will meet the study criteria and be interested in trying to quit [10], and 50% or more of these patients will be interested in participating in the study [3,9,10,15]. Thus, if we underachieve our recruitment goal by half we will still meet our recruitment target.

We expect a high follow-up rate for six- and twelve-month assessments. Studies of KPNW and OHSU patients typically achieve follow-up rates of 85% and above for clinical studies [10,15-17]. Each site employs experienced recruitment staff who use electronic tracking systems that collect and maintain up-to-date scheduling and contact information, notification of upcoming follow-up encounters, and web- and phone-based queries for missing addresses.

#### *EMR prescreening*

Each morning, information for admitted patients is captured from the EMR and downloaded into an electronic report used for prescreening. Patients are excluded if they are currently under age 18, have no recent smoking history, are located in one of the excluded units, or have already been screened for the study. Since the number of eligible smokers per day is likely to exceed the capacity of study staff, an algorithm is used to create a daily contact list that objectively assigns patients a contact order (1-n) for in-person screening and recruitment. The algorithm also incorporates recruitment targets for Hispanics, racial minorities, and females. Once the contact list is generated, the TTS reviews electronic records for additional exclusions. Decision making for physical and cognitive exclusions is guided by nurse and physician scope of practice at each site. The remaining prescreened eligible patients are contacted by the TTS for additional screening and recruitment is conducted as part of a tobacco-cessation consult. Patients on the contact list who are not seen by a TTS are re-prioritized the next day if they are still hospitalized.

#### *In-person screening and baseline assessment*

All patients contacted and willing to discuss tobacco use are provided a brief tobacco-cessation consult (see usual care description below). The TTS conducts face-to-face screening to confirm eligibility, assess interest in participation, conduct informed consent procedures, complete a baseline assessment, and randomize participants to the AR + IVR or UC groups.

### Randomization

Randomization is conducted using a selection and documentation procedure that ensures balanced enrollment over time, blinds the TTS to the assignment, and prevents postrandomization assignment changes. Each site randomly assigns 450 patients on a 2:1 ratio (300 AR + IVR: 150 UC) using secure, preprinted, sequentially numbered randomization envelopes. The study statistician developed a randomization algorithm using a predetermined randomization sequence with permuted blocks of randomly varying size. The block size is masked from all except the data coordinating center (DCC) statistician and programmer. This strategy ensures balanced enrollment over time and eliminates the ability of the TTS to guess assignment prior to randomization.

The randomization algorithm matches group assignment to a four-digit randomization number, where the first digit is the site ID (KP = 1; OHSU = 2) and the last three digits are the randomization sequence number. The randomization number is printed on a label and affixed to the outside of each randomization envelope. Inside each envelope are two identical self-adhesive labels printed with the randomization number and treatment arm assignment. The labels are used to confirm and track randomization. The envelopes are placed in a box in ascending order. The treatment arm assignment is not discernable from the outside of the sealed envelope.

Once a patient is prescreened eligible, the TTS selects the next randomization envelope in sequence, along with other patient enrollment forms, and proceeds to the patient’s room. After the eligible patient provides informed consent and completes baseline assessment, the TTS opens the randomization envelope and records the participant’s assignment. At the KP site, the TTS affixes one of the randomization labels to the site’s randomization log, and then records the date and time of randomization, the participant ID number, and the TTS’ initials on the log. A second label is affixed to the enrollment checklist form before transmission to data entry staff. At the OHSU site, the TTS opens the randomization envelope, places one of the randomization labels in the patient’s research chart, records the date and time of randomization, participant ID number, and the TTS’ initials. A second label is affixed to the enrollment checklist before transmission to KP data entry staff.

Randomization fidelity is monitored regularly by comparing information from the randomization log, prescreening priority list, and the consult and enrollment checklists. The fidelity review process involves comparisons of participants’ randomization dates and times, group assignments, and predetermined randomization numbers. Any unexpected deviation will be reported to the study principal investigator. Participant assignment fidelity is maintained during the study by restricted access to the file service containing randomization data to the study statistician and data analyst. Study investigators and follow-up staff are blinded to treatment group data.

All randomized patients will be included in the intention-to-treat analysis of the primary outcome. We use a 2:1 assignment strategy to increase the number of AR + IVR recipients in order to enhance analyses of an expected dose-effect on outcome, particularly for AR + IVR participants. The 2:1 assignment strategy also fits our respective health system’s interest in maximizing the potential treatment benefits to study participants, which is important to increase cooperation among institutions in the study and helps build organizational interest in improving cessation services. We do not stratify recruitment on population characteristics other than race-ethnicity and gender. Stratification on factors determined during the patient interview, such as nicotine dependence, is not practical in a busy hospital setting. The additional complexity would add time to the patient encounter, potentially disrupt patient care, and jeopardize recruitment. Any variation in patient factors affecting quitting will be evaluated in the statistical analysis. Combining data across all CHART studies will also aid in the evaluation of quit predictors across sites.

### Study interventions

At each hospital, the patient’s tobacco-use history is assessed at bedside by an admitting nurse and recorded in the EMR. A TTS at each hospital reviews the EMR for initial study eligibility (prescreening) prior to conducting a bedside consult and screening visit. All study participants receive a brief (10-minute) bedside tobacco-use evaluation and cessation consult that serves as the usual care condition. All intervention service and medication use, both as an inpatient and after discharge, will be tracked for all study participants. Internal evaluation data indicate the use of outpatient cessation services and medications following hospital discharge was extremely rare (<1% of discharged smokers) among KPNW and OHSU members.

We expect that about 15% of UC recipients will be smoke-free at six months. Unpublished evaluation data for hospitalized KPNW members and OHSU consult recipients suggest about 15% of discharged patients will remain abstinent six months after discharge. Stevens *et al*. (2000) [10] reported a 14.6% quit rate for KPNW hospitalized smokers receiving an intervention similar to our usual care. We estimate that 23% of AR + IVR recipients will be abstinent at six months, given an odds ratio of 1.65, compared to UC recipients [3].

#### *Inpatient tobacco cessation consult (usual care)*

The usual care (UC) intervention provided by the TTS involves a tobacco-use and quit history assessment, discussion of the health consequences of tobacco use and benefits of quitting, and tailored discharge treatment recommendations based on the patient’s tobacco history and personal circumstances. Tobacco-use assessment includes the types of tobacco used, amount used per day, age at initiation, and use by other family members. Quit history includes the number of past quit attempts made, how long ago they last tried to quit, and their experience with using cessation programs and medications. If they used medications, patients are asked about the types of medications used (nicotine replacement therapy [NRT], bupropion, varenicline), how they liked it, and if they experienced any side effects. If appropriate, a tailored discussion is provided of the relationship between tobacco exposure and the patient’s diagnosis, and the health benefits from remaining abstinent. Patients are encouraged to remain abstinent after discharge and provided printed information about available outpatient resources.

UC recipients are provided printed information and a brief overview of existing counseling programs and FDA-approved cessation medications that are typically covered by the patient’s health insurance. UC patients are informed about how to access available services on their own, that is, the TTS does not actively assist the patient in enrolling in a program or ordering medications.

At KPNW, patients access outpatient cessation services and medications through the Health Education Services (HES) department. Patients can enroll in telephone-based and in-person counseling programs, or an interactive web-based program, by contacting HES on their own. Available medications include over-the-counter NRT (transdermal patch, gum, lozenges) and prescription bupropion and varenicline. KPNW patients are charged a small copayment for the services they select, and must enroll in an approved counseling program in order to receive medications at copay. Patients can purchase cessation medications at discharge, typically through the patient’s attending physician; however, the patient will be required to enroll in a counseling program through HES in order to get medications at copay.

At OHSU, patients are provided information about alternative counseling programs and available NRT products (the patch, gum, lozenges, nasal sprays), bupropion, and varenicline that are typically covered by insurance. Patients are encouraged to call their insurance provider to determine cessation program coverage. Patients with Medicare, Medicaid, or no insurance coverage are given information about telephone counseling provided through the state quit line (1-800-QUIT-NOW).

#### *AR + IVR Intervention*

AR + IVR assigned patients are assessed for interest and willingness to enroll in stop-smoking services following discharge. Those who express interest are helped to enroll in available programs before leaving the hospital and staff initiate discharge orders for cessation medications. At KPNW, available programs include alternative telephone counseling programs, individual and group classes, and an interactive web-based program. All programs, except the web-based program, have been shown to be effective at helping smokers quit [9,15,18]. KPNW currently offers nicotine replacement therapy (transdermal patches, lozenges, gum), bupropion, and varenicline. OHSU participants will be offered enrollment in the Oregon Tobacco Quit Line (OTQL) and provided information about available resources and medication options. Information about study enrollment and recommended options for postdischarge cessation treatment will be sent to each patient’s primary source of health care via internal EMR notes or external fax or electronic notes. At both sites, the out-of-pocket costs for enrolled services are determined by participants’ insurance coverage.

#### *IVR follow-up calls*

After discharge, AR + IVR recipients receive four IVR follow-up calls, at days 4, 14, 28, and 49 using Eliza Corporation’s IVR system [19]. The call receipt window will be +4 days for the initial call, and ±4 days for each subsequent call. Participants are prompted for information on current smoking status, cessation program enrollment status, and assessment of cessation medication use. Patients also receive brief, tailored, supportive messages to help them stay off cigarettes. These messages include encouragement to enroll in cessation programs, use cessation medications, or to speak to their doctor to determine the best course of treatment. IVR alerts are generated when a patient indicates that he/she would like to speak to an OTQL quit coach or KP health coach during the call. Study staff members facilitate a callback from the respective coach.

#### *Intervention fidelity*

All research staff will be trained and certified in good clinical practice. Prior to study implementation, all TTSs are trained in how to deliver tobacco-dependence treatment to hospitalized patients based on the OHSU model. TTSs are also monitored during initial piloting and will participate in ongoing case management discussions. Other staff will receive appropriate training in use of the study’s electronic data management system at each site and in coding rules to complete the forms properly.

We monitor intervention fidelity by tracking inpatient cessation service delivery, assisted referrals to outpatient counseling and medications, notifications to primary care providers, and EMR-documented and self-reported cessation service use at follow-up. Inpatient counseling is tracked by documenting TTS-patient interaction time, and by documenting cessation topics discussed during the consult. A treatment form is used to record TTS-patient discussion of tobacco use and quitting history, past cessation service and medications use, current inpatient NRT dispenses and comfort, risk factors for maintaining abstinence, medical contraindications for medication use after discharge, interest in remaining abstinent after discharge, and outpatient treatment recommendations provided. The intervention protocol and treatment form do not specify that a particular topic order must be followed by the TTS. Instead, the form is completed based on topics discussed. Treatment data are recorded and monitored for completeness and additional training conducted as needed.

For study participants randomized to the AR + IVR condition, we obtain documentation of assisted referral outcome (acceptance/refusal of referral, discharge medication ordered), primary care/usual care provider notification completed, and IVR enrollment. Outpatient intervention use for all enrolled participants is obtained during the study period from EMR records and from patient self-reports at follow-up. The IVR intervention is provided via a standard IVR automated calling system for participants at both sites. The content for each call is standard for both hospitals, with some site-specific tailoring. Each call uses branching logic based on information provided from the previous call to gather more accurate and personalized information.

### Safety monitoring plan

Behavioral counseling and FDA-approved cessation medications are considered low-risk interventions for tobacco-use treatment. Adverse events (AEs) associated with medication use are well known, for example, skin rashes, irritability. In some instances, serious adverse events (SAEs) linked to increased risk of suicide have been documented among bupropion and varenicline users [20-22]. This trial involves patients hospitalized for potentially serious health conditions, which increases the risks of re-hospitalization or death.

This study follows the CHART DSMB recommendations, and KPNW and OHSU IRB policies, for tracking and reporting SAEs and AEs. An adverse event tracking and reporting system is used to monitor expected and unexpected SAEs and AEs reported by participants at six- and twelve-month follow-up. The principal investigator or medical monitor will review reported events to determine if they are study-related. Unexpected or potentially study-related AEs and SAEs, including confidentiality breaches, will be reported to the local IRBs within forty-eight hours of identification, and to the DSMB within seven days. SAEs and AEs that are not study-related will be reported to the DSMB every six months and to the local IRBs every twelve months.

### Study measures

Intervention delivery and data collection occur at an inpatient tobacco-cessation consult and screening/recruitment visit, postdischarge IVR telephone calls (AR + IVR only), in-person follow-up interview at six months postrandomization, and during a telephone interview at twelve months. Procedures are standardized as much as possible between the two settings in order to avoid confounding differences in recruiting methods due to site differences. Furthermore, the primary and secondary outcomes for this study have been harmonized with other CHART studies following recommendations of the CHART DSMB.

#### *Primary outcome*

The primary outcome for the I-TSAR study is self-reported thirty-day abstinence from cigarettes at six months postrandomization for AR + IVR compared to UC. Data on days since the last cigarette (even a puff) are collected from participants at six- and twelve-month follow-up assessments. The number of days since the last cigarette was smoked allows calculation of seven-day, thirty-day, and continuous abstinence as secondary outcomes. Biochemical validation of self-reported abstinence is obtained at six months and reported as an additional outcome. All participants are given an exhaled carbon monoxide assessment at the six-month in-person assessment using a Bedfont Smokerlyzer Carbon Monoxide Monitor (Bedfont Scientific Ltd., Maidstone, Kent, UK) [23]. Self-reported abstinence of ≥7 days is verified by a CO level ≤8 parts per million (ppm). For comparison with other CHART studies, self-reported quitting is confirmed using a salivary cotinine (NicAlert) test strip (Nymox Pharmaceutical Corp., Hasbrouck Heights, NJ, USA). Salivary cotinine measurement of ≤50 ng/ml confirms abstinence [24]. Self-reported quitters using nicotine replacement products at the time of the six-month assessment will be considered as ‘confirmed quitters’ by a CO level of ≤10 ppm.

#### *Power*

A 2:1 ratio of AR + IVR to UC for 900 randomized participants will provide adequate power to detect an eight percentage point difference in thirty-day point prevalence abstinence at six months (our primary outcome), assuming an unbiased method of assigning an outcome to nonrespondents. The unbalanced design requires 864 participants to achieve 80% power, with two-tailed α set at .05, to detect a difference in thirty-day abstinence of eight percentage points between treatment groups. We expect the UC group will achieve a 15% abstinence rate, based on analyses of EMR data (unpublished) for similar populations at each site. An odds ratio of 1.65 for patients with >30 days of follow-up [3] results in a 23% expected abstinence rate for the AR + IVR group. The power calculations were conservative [25-28] and no continuity correction was applied. Rounding up to 900 total participants added an additional measure of conservatism.

#### *Additional tobacco measures*

For participants reporting having smoked during the follow-up period, we assess amount smoked per day, and the first two consecutive days smoked postdischarge, to calculate relapse curves by group assignment. We also collect baseline and follow-up information about other tobacco use, including cigars, pipes, bidis, hookahs, and smokeless tobacco to control for substitution. We ask about other household smokers and indoor smoking rules.

#### *Intervention dose*

We collect inpatient and outpatient smoking cessation counseling and medications provided and used by study participants from the initial inpatient consult through 12-month follow-up. Measures collected are used to assess treatment fidelity and to assess an intervention dose response on the primary outcome. Inpatient treatment measures include: TTS counseling time and content checks; medication dispenses; quit materials provided; and inpatient referrals to outpatient services (counseling programs and medication orders). These data are collected from TTS and EMR data sources. Outpatient treatment measures include: counseling program enrollment and participation, such as telephone quitlines, in-person individual and group programs, and web-based programs; FDA-approved prescription and over-the-counter medications such as buproprion, varenecline, and nicotine patches, gum, lozenges, and sprays. Medications provided and used, including combination therapy, will be assessed from EMR records and participant self-report.

#### *Intervention resource costs*

We collect tobacco cessation intervention delivery costs for study participants from the initial inpatient consult through 12-month follow-up. Cost data are obtained to support analyses from participant, health system/insurer, and societal perspectives assuming an opportunity cost approach to resource valuation. Source of payment for each expense will be used to assign costs. Intervention costs we use in the study include: TTS inpatient prescreening time; TTS patient contact time; inpatient medication dispenses; outpatient cessation counseling and medication costs; printed quit materials; IVR follow-up costs; patient travel expenditures for cessation treatment; and patient lost time from work for treatment and hourly earnings. Health system costs we use include: TTS and other staff training time; program management time; new materials development and supplies; and internal feedback reporting.

#### *Health-care utilization and expenditures*

We collect health-care utilization data beginning with the initial hospitalization through 12-month follow-up. Data include: encounters by type (primary care, emergency department, urgent care, hospital admissions); length of hospital stay (initial and subsequent hospitalizations); discharge diagnoses (ICD-9); procedures (CPT-4); pharmacy dispenses and NDC codes; diagnostic-related groups; and medical records data for health-care expenditures (actual costs based on relative-value units and paid claims; retail costs for medications) by source of payment. Data are used to assess differences in utilization and expenditures by treatment group.

#### *Health-related quality of life*

We obtain patient responses from the EuroQual (EQ5D-5L) [29-31] at baseline and six- and twelve-month follow-up for assessing changes in health-related quality of life for AR + IVR versus UC participants during the study period. Responses are combined with remaining life expectancy data for current and former smokers [32] to estimate differences in expected quality-adjusted life years saved by treatment group.

#### *Other patient information*

Age, sex, ethnicity, race, height, weight, marital status, educational attainment, annual income, insurance coverage, time after waking before first cigarette, confidence for successful quitting, Patient-Health Questionaire-2 depression screen, and the Audit-C alcohol screen, are collected at baseline for primary and secondary outcome analyses. Weight is collected at follow-up.

### Data analysis

This section describes the methods used to evaluate the specific aims of the I-TSAR study. The primary aim for this study has been harmonized with the other NIH-funded CHART collaborative group studies and approved by the CHART DSMB.

#### *Specific Aim 1: estimate self-reported thirty-day abstinence at six and twelve months for patients assigned to receive inpatient AR + IVR compared to usual care*

Based on KPNW and OHSU member data and meta-analyses of effectiveness of hospital cessation programs with ≥1 month postdischarge follow-up, we expect about 15% of usual care patients and 23% of AR + IVR participants will be abstinent six months following discharge. The primary analysis assumes that nonrespondents are smokers.

The treatment effect on the primary outcome (and other binary outcomes) will be evaluated using logistic regression, logit[Y_ik_] = β_0_ + β_1_X_k_ + e _ik_ (Model 1), where Y_ik_ is the binary indicator of the observed binary smoking status for the i^th^ patient (i = 1,. . .,n_k_) within the k^th^ treatment condition (k = 1,2), X_k_ is the fixed effect of the k^th^ condition, that is, intervention or control, and β_0_, β_1_ are parameters to be estimated. Any difference between the predicted and observed values is left to the residual error (e _ik_) in this model. Multiple regression methods available in SAS PROC Logistic [33] will be used to estimate the parameters of this generalized linear model. The intervention fixed effect will be tested using the score *χ*^2^ test.

Model 1 specifies only one source of systematic variation among the trial conditions, C_k_. Any factor other than the intervention that favors one condition over the other serves to bias the estimate of the intervention effect. Since this is a randomized trial, there are no differences expected between treatment and control arms in the distribution of covariates. However, there might be unmeasured factors at the various sites that might result in varying effect sizes between the two arms. These factors are potentially confounded with site. In the adjusted analysis, site, interaction between site and treatment, and measured covariates are added to the analysis to reduce confounding and to improve the precision of the estimate of the intervention effect. Covariates can be measured at the site or patient level. Covariates measured at the group (site) level and patient level are distinguished as **G** and **M**. With covariates at two levels and patients nested within sites, we shift to a two-level generalized linear model with logit link. Level 1 involves patient-level variables: $\text{logit}\;\left[Y_{\text{ick}}\right]=\beta_{0c}+\beta_{1c}X_{k}+\beta_{2c}M_{i}+e_{\text{ick}}$, where **M**_i_ is a vector of patient-level covariates (bold indicates a vector throughout this section), *β*_0c,_*β*_1c_, and ***β***_2c_ are patient-level parameters that are allowed to vary over sites, and c = 1, . . ., C clinical sites, and e_ick_ is the residual.

The level-1 model implies three level-2 equations to model variation between sites in patient-level parameters and adds the effect of site-level covariates:

where γ_0._, γ_1_., γ_2_ are parameters to be estimated, Sc is the vector of c-1 site indicators, Gc is the vector of site-level covariates, and u are residual terms. The resulting combined two-level model is of the form:

(1) $$\text{logit}\;\left[Y_{\text{ick}}\right]={ϒ}_{00}+{ϒ}_{01}Sc+{ϒ}_{02}Gc+u_{0c}+\left[{ϒ}_{10},+,{ϒ}_{11},S,c,+,{ϒ}_{12},G,c,+,u_{1c}\right]X_{k}+\left[{ϒ}_{20},+,{ϒ}_{21},S,c,+,{ϒ}_{22},G,c,+,u_{2c}\right]M_{i}+e_{\text{ick}},$$

that simplifies to (Model 2):

(2) $$\text{logit}\left[Y_{\text{ick}}\right]={ϒ}_{00}+{ϒ}_{01}Sc+{ϒ}_{02}Gc+\left[{ϒ}_{10}+u_{1c}\right]X_{k}+{ϒ}_{11}S{\text{cX}}_{k}+{ϒ}_{12}G{\text{cX}}_{k}+\left[{ϒ}_{20}+u_{2c}\right]M_{i}+{ϒ}_{21}ScM_{i}+{ϒ}_{22}GcM_{i}+u_{0c}+e_{\text{ick}},$$

where combined effects, for example, **S**cX_k_, indicate an interaction. Not shown here are the multiple slope coefficients on the vectors of **M** and **G** covariates. We will test the assumption of linear relationships between quantitative covariates and the logit of smoking status by adding quadratic terms to Model 2. The test of homogeneity of covariate regression slopes across conditions and sites is the test that the associated parameter (for example, γ_11_) is 0. When the covariates were measured at baseline, this is a test of whether the variable moderates treatment response. This could provide useful information on subgroups of smokers for whom the intervention is more or less effective.

#### *Covariates and effect modifiers*

Group-level covariates considered for the analysis include site (KPNW and OHSU), insurance type, inpatient care unit, smoking cessation program used, and medications used. Individual-level covariates include age, sex, ethnicity and race, marital status, amount smoked per day, time to first cigarette, stage of readiness, depression screen positive, alcohol abuse screen positive, socioeconomic status (income, education), and other smokers in the household. Though power will be limited, we will report treatment effects for race/ethnicity categories and by sex.

Secondary analyses will use self-reported seven-day and continuous abstinence at six and twelve months, biochemically confirmed seven-day abstinence at six months, number of quit attempts during the prior year (modeled as Poisson), and amount smoked among continuing smokers (modeled as Poisson).

#### *Specific Aim 2: estimate the dose effect on smoking abstinence at six and twelve months for the AR + IVR compared to usual care*

Variation in outpatient service use is used to assess a dose–response effect for cessation services. We test the hypothesis in two ways using the model developed for Specific Aim 1. First, we will include only AR + IVR recipients and redefine X as a multilevel ordinal variable representing intervention dose (D_r_) ranging from no postdischarge intervention to full intervention. The number of levels to D_r_ will be determined using a rank scoring algorithm that includes cessation service program enrollment and completion, cessation medications, and number of IVR follow-up calls received. Additional inputs will be considered based on actual study data. Second, we will rerun the analysis including control group participants with a dose score using the same method. For each analysis, we will conduct trend tests and assess the presence of a dose threshold.

#### *Specific Aim 3: estimate total and mean costs per participant for AR + IVR and UC recipients, and, the incremental cost-effectiveness of AR + IVR at six and twelve months compared to UC from health plan/insurer and societal perspectives*

We hypothesize that AR + IVR is a cost-effective strategy at six and twelve months for smoking cessation compared to UC from societal, health plan/insurer, and individual patient perspectives. Assuming the intervention is effective, we calculate the incremental net cost per additional quit for AR + IVR recipients compared to UC, and incremental costs per quality-adjusted life-years saved (QALYs).

Total intervention costs and costs per participant for AR + IVR and UC are evaluated. All resource costs used to implement and deliver the inpatient and postdischarge follow-up services, counseling programs, medications, and IVR calls are included in the cost calculations. The incremental net cost is calculated per additional quit for AR + IVR participants compared to UC participants, and incremental costs per quality-adjusted life-years saved (QALYs) [34,35]. Program cost analysis (PCA) is used to assess the actual economic, or opportunity, costs incurred to produce the outcome observed from the AR + IVR and UC interventions. The value and the incremental resources required for the intervention for the health system/insurer and societal perspectives are identified. Individual-level cost data are collected to enable variance estimation for the average cost estimates. Total intervention costs and costs per participant for AR + IVR and usual care recipients are estimated.

Abstinence and cost data are used to calculate the incremental net cost per additional quit for AR + IVR participants compared to UC participants. Incremental total costs and cost per participant, by payment source are reported. Incremental cost-effectiveness ratios (ICERs) are measured as: a) incremental intervention costs per incremental quit from health plan/insurer and patient perspectives; b) incremental total costs (intervention plus health care expenditures during follow-up) per incremental quit from health system/insurer and patient perspectives; c) incremental intervention costs per incremental quality-adjusted life-year (QALY) saved from a societal perspective; and d) incremental total costs (intervention plus health-care expenditures during follow-up) per incremental QALY saved from a societal perspective. QALYs for each intervention arm are estimated using life expectancy data and responses from interviewer-administered EQ5D-5L.

#### *Specific Aim 4: evaluate health care utilization at twelve months for AR + AVR and usual care*

We measure health-care utilization for AR + AVR compared to UC by the mean number of combined outpatient and inpatient encounters (visits) per participant during follow-up. This aim is addressed using participant survey responses and medical records (electronically for those receiving care at KPNW and OHSU facilities). For AR + IVR compared to UC, we measure health-care utilization by the mean number of combined outpatient and inpatient encounters (visits) per participant during follow-up. This aim will be addressed using participant survey responses and medical records (electronically for those receiving care at KPNW and OHSU facilities). The functional form of models 1 and 2 for the primary aim will be restated within a Poisson regression model structure and analyzed using SAS PROC GENMOD, specifying a log link function and Poisson distribution.

Health-care utilization data are regularly characterized by nonnormal distributions. While zero values are not expected for visits in the study population, there may be substantial variation in the number of visits completed. Depending on the number of inpatient visits, analysis of the number of inpatient days separately from outpatient visits may be conducted. If the variance is greater than the mean, we will control for the impact of overdispersion by including a term in the model for the unobserved heterogeneity of each i^th^ observation. If data allow separate modeling of inpatient days, the large number of zero stays will require specifying a zero-inflated negative binomial model.

#### *Sensitivity and threshold analyses*

Sensitivity of cost-effectiveness ratios to key parameters: intervention acceptance, intervention costs, abstinence rate, discount rate, pharmacotherapy use and costs, and QALYs saved are measured. Threshold analyses for key parameters are used to identify input levels that alter conclusions. The cost-effectiveness analysis above reflects a deterministic approach using standard univariate and multivariate sensitivity analyses to test the robustness of the results. Based on the results of these analyses, probabilistic sensitivity analyses will be considered, but only if the standard sensitivity tests are unclear or not informative [36-39].

### Missing and incomplete data

Nonresponse is defined as when a participant is lost to follow-up or refuses to answer questions about their smoking status. For our primary hypothesis test, we assume participants lost to follow-up are smokers. Depending on actual follow-up rates achieved, the results may be presented using recommended new strategies [40] to deal with missing data. Characteristics associated with nonresponse in smokers are used with SOLAS and SAS programs for implementing multiple imputation procedures. A complete record of subject assignment and attrition will be included in published reports of this trial. For subject attrition, the type of attrition (that is, ‘lost to follow-up’, ‘deceased’, and ‘refused to continue participation’) is monitored. An attempt will be made to identify the causes of missing data. If smokers are more likely than nonsmokers to be lost to follow-up or to be refusals, then nonresponse is ‘nonignorable’ (that is, nonresponse is related to the value of the smoking variable that would have been observed).

A large proportion of the study population will have had a major adverse health event. Thus, the number of patients deceased at follow-up may be higher than is typical in other smoking cessation studies. Patients deceased before follow-up will be excluded from the primary outcome analyses. We will identify the number of deceased patients in the consort table. We may include deceased patients in the economic evaluation because of the high cost of end-of-life care. In the cost-effectiveness analyses, we will evaluate deceased patients as smokers (treatment failures) and assign them a health-related quality of life value of 0. We will also use analytic methods that account for censoring for patients with limited health-care utilization data. For example, for KPNW participants who leave the plan after several months, and for whom we are unable to obtain medical records and expenditures for the entire follow-up period, will have their encounter (visit) and expenditure data for the entire follow-up period annualized based on the proportion of the year for which they were enrolled in the heath plan.

## Discussion

Effective and cost-effective approaches to bridging inpatient and outpatient tobacco dependence treatment can be an important part of health system efforts to improve patient health and reduce future health-care costs. This study provides estimates of the health impacts, and economic impacts, of a practical approach to help smokers remain abstinent after hospital discharge. Our intervention protocol, providing brief inpatient cessation counseling, assisted referral to effective outpatient counseling and medications, and IVR follow-up over a seven-week period, is in line with the findings from a recent Cochrane meta-analysis showing that smokers with four or more weeks of cessation support after discharge were 1.65 (CI 1.44–1.90) times more likely to quit successfully after a year compared to those without support [3]. We expect to see a similar relationship between the AR + IVR and UC groups, but also expect a dose–response for participants based on the number of cessation services used (counseling sessions and medication days). Our 2:1 randomization strategy will allow us to evaluate the relationship between intervention dose and outcome.

The setting for this study provides an opportunity to compare study outcomes in two different health systems, a large comprehensive managed care organization and a large academic hospital. Since KPNW is a closed system, we expect the integration of inpatient and outpatient care to be more efficient there because of the ease of access to patient medical records and streamlined communications between inpatient medical staff, pharmacy staff, and outpatient care providers. Determination of plan benefit levels (for example, copays, medication availability) is also readily available. OHSU has a diverse patient population. Linking inpatient and outpatient care will be more complicated for OHSU participants since many patients do not have a regular health-care provider or are uninsured and receive outpatient care from providers or clinics outside of the OHSU system. Informing these providers about their patients’ cessation attempts will be more difficult. Also, access to services may vary based on differences in insurance coverage. We will evaluate whether these system differences affect treatment outcomes.

The ultimate goal of this study is to permanently integrate a practical tobacco cessation program into the participating health-care systems. This effort requires an understanding of the economic, logistical, and organizational challenges faced by each organization. To be effective in the real world, an intervention must integrate easily into the existing clinical workflow and become an integral part of the health plan’s normal functioning. To accomplish this, we use Bracht’s five-stage community organization model [41] to guide our support-building activities within each organization. We identify key stakeholders at each institution who are working with study staff to ensure that the intervention protocol fits the needs, resources, and values of the organization. We will share intervention recruitment, delivery, and acceptance measures with stakeholders using periodic clinic feedback reports. Performance measures relevant to each institution will help the program make the transition from a novel intervention to standard care at each organization. In addition, the I-TSAR investigators will provide data on intervention reach, effectiveness, and cost analyses to support financial decisions and commitments.

## Trial status

The I-TSAR study is currently recruiting participants. Recruitment began in November 2011, and is expected to conclude in May 2013.

## Abbreviations

AR + IVR: assisted referral plus interactive voice recognition; CHART: Consortium of Hospitals Advancing Research on Tobacco; DSMB: Data Safety Monitoring Board; DCC: data coordinating center; EMR: electronic medical record; ICER: incremental cost-effectiveness ratio; IRB: Institutional Review Board; I-TSAR: Inpatient Technology-Supported Assisted Referral; KPNW: Kaiser Permanente Northwest; NIH: National Institutes of Health; NRT: nicotine replacement therapy; OHSU: Oregon Health & Science University; OTQL: Oregon Tobacco Quit Line; QALY: quality-adjusted life year; (S)AE: (serious) adverse event; TTS: tobacco treatment specialist; UC: usual care.

## Competing interests

The authors declare that they have no competing interests.

## Authors’ contributions

All listed authors substantively contributed to the study design or conduct, and in the development of this manuscript. JF, RM, and LW contributed to the study conception, design, and study funding. JF, RM, LW, and KA developed the inpatient treatment and assisted referral protocol. LW, KF, SL, and JM contributed to data model design, acquisition and management, and quality control. JF, RM, LW, and KA assisted with health system liaison. All authors reviewed and approved the final version of the manuscript.

## Authors’ information

Jeffrey L. Fellows, PhD, and Richard Mularski, MD, are investigators and experienced tobacco control researchers at the Kaiser Permanente Center for Health Research. David Gonzales, PhD, and Wendy Bjornson, MPH, lead the OHSU Smoking Cessation Center and are experienced tobacco control researchers.
